# Items

**Published:** 1861-04

**Authors:** 


☞ In the American Journal of Pharmacy, as gleaned from German Journals, we find the following useful items:

*Reaction of Strychnia.*—The solid strychnia when mixed with solid iodic acid, or iodate of potassa and a drop of strong sulphuric acid, assumes, according to X. Lauderer, on the application of a gentle heat, a beautiful violet color, gradually changing to red brown, remaining unchanged for many days. The thick liquid shows after a few hours a beautiful iridescence continuing for several days.

*Silicon in iron.*—The black residue left after dissolving crude iron in muriatic acid, was found by Schafhäutl to evolve hydrogen with ammonia. Woehler has proved it to contain oxide of silicon.—(Ann. d. Chem, and Ph. xxviii. 374.)

*Panis Laxans*, or laxative cake, is prepared by painting the underside of small biscuite with an alcoholic solution of jalap-resin, and covering the place with a thin layer of a mixture consisting of beaten albumen, sugar and a little tragacanth; each cake to contain 2 gr. of resin. The dose as a mild laxative is 2 or 3 cakes for a grown person, 1 for a child from 6 to 8 years, &c.—(Pharmac. Centralhalle i. No. 12.)

*Tin in vinegar.*—Investigations caused by the State authorities in Potsdam have developed the fact, that pure vinegar carried in bright tin measures from the cellar to the store contained a small quantity of tin, which increased on standing and was considerable after boiling. Tin measures may probably be frequently the cause of the deleterious effects of vinegar, looked for in other circumstances.—(Arch. d. Med. Ges.—Arch. d. Pharm. cli. 115.)

*Suppositories.*—Dr. Pfeiffer prepares them as follows: cacao-butter or suet is fused with ⅛ white wax and moulded. Immediately before hardening, a tube is pressed through the base towards the middle, producing a channel which is to be filled with the medicinal substances desired, after which the aperture is closed by butter of cacao. Thus they become more active, and may be kept all ready except the filling.—(Arch. d. Pharm. cli. from Journ. des Connaiss. Méd. et Ph.

*Creasotum chloroformatum.*—The following or a similar mixture has long been in use in France: Creasote 1 p., chloroform and alcohol of each 2 p., by weight. (Ph. Centralhalle i. 1.)

*Aqua St. Johannis.*—The following mixture is employed in Southern France as a vulnerary, diminishing suppuration, and being useful in all cases where spirit of camphor and lead water is indicated: sulphate of zinc ʒj., sulphate of copper ℈j., water ℥xxxvj., dissolve and add tincture of saffron ʒij., spirit of camphor ℥ij., (Pharm. Cent. Halle i. No. 6.)

*Glycerine in incipient dysentery* is employed by Dr. Daude in the form of clysters, each composed of glycerine ʒiv. and mucilage of linseed ʒxx. At the same time, of the following mixture a tablespoonful is given every hour: glycerine ℥jss. water and orange flower water each ℥iv. (Ph. C. H. I. No. 6.)

